# Myocarditis in Hypertrophic Cardiomyopathy: Incidence and Contribution to Disease Progression and Outcome

**DOI:** 10.31083/RCM28234

**Published:** 2025-04-17

**Authors:** Yulia Lutokhina, Nasiba Seifatova, Polina Chernova, Natalia Kireeva, Roman Komarov, Marina Vukolova, Sergey Pirozhkov, Ekaterina Yantikova, Nargiza Babakulova, Boris Tlisov, Andrey Dzyundzya, Beatrice Volel

**Affiliations:** ^1^Moscow State Medical University of the Ministry of Health of the Russian Federation (Sechenov University), 119991 Moscow, Russian Federation

**Keywords:** HCM, hypertrophic cardiomyopathy, primary myocardial hypertrophy, myocarditis, inflammatory cardiomyopathy, myocardial biopsy, immunosuppressive therapy

## Abstract

**Background:**

Myocardial diseases such as myocarditis and cardiomyopathies are clinically important and can cause complications such as heart failure and arrhythmias, which increase the risk of death. The combination of myocarditis with cardiomyopathy is difficult to diagnose because their manifestations often overlap, and multiple myocardial diseases are usually not included in the diagnostic search. Hypertrophic cardiomyopathy (HCM) is the most common cardiomyopathy; however, few studies have examined the combination of myocarditis and HCM, thereby highlighting the importance of this problem. This article aimed to analyze the influence of myocarditis on clinical features and outcomes in patients with HCM.

**Methods:**

A literature search was performed using PubMed and the Scientific Electronic Library eLIBRARY.ru databases. Relevant studies, published until November 2023, were analyzed in detail. Studies were selected in accordance with the Preferred Reporting Items for Systematic Reviews and Meta-Analyses (PRISMA) standards.

**Results:**

Twelve studies (three original articles and nine clinical cases) were isolated from a total cohort of 1504 publications and were included in the study. The prevalence of myocarditis in HCM ranged from 23.5% to 46.7%. The presence of concomitant myocarditis in patients with HCM was associated with heart failure progression, worsening of ventricular arrhythmias, and an increased risk of sudden cardiac death.

**Conclusions:**

The incidence of myocarditis in HCM is high. Early detection and treatment of myocarditis in patients with HCM can slow the progression of heart failure rhythm disturbances and improve the disease prognosis.

**The PROSPERO Registration:**

The systematic review was registered in the International Prospective Register of Systematic Reviews PROSPERO (CRD42024499672, https://www.crd.york.ac.uk/PROSPERO/view/CRD42024499672).

## 1. Introduction 

Recently, myocardial diseases have attracted the attention of the cardiology community. Due to improvements in diagnostic approaches, such as the widespread availability of cardiac magnetic resonance imaging (MRI) and DNA diagnostics, their prevalence in cardiovascular disease has increased significantly compared to previous years [1]. Although myocardial diseases are not as common as coronary artery disease or heart defects and hypertension in the general population, myocardial diseases are clinically significant and can lead to serious complications such as heart failure and arrhythmia, which increase the risk of death [2]. The most common forms of myocardial diseases include myocarditis and cardiomyopathies. The relationship between these conditions has recently been a topic of great interest, and the modern cardiology community is actively discussing the combination of these diseases in patients. Two main theories exist about how these diseases may occur simultaneously in the same person. According to the first theory, in primary cardiomyopathy, the genetically compromised myocardium may provide a favorable background for viral infection or autoimmune reaction of the body [3, 4, 5]. Conversely, the second theory suggests that myocarditis initiates an abnormal genetic program that leads to the development of cardiomyopathy [6].

The diagnosis of a combination of cardiomyopathy and myocarditis can present significant challenges for healthcare professionals. Firstly, the symptoms of these conditions often overlap, making differentiation difficult. Secondly, physicians frequently limit themselves to a single diagnosis without including a combination of several myocardial diseases in their diagnostic search. Case reports have documented the co-occurrence of myocarditis and various forms of cardiomyopathy, including arrhythmogenic right ventricular cardiomyopathy [7, 8], dilated cardiomyopathy [9, 10, 11], and left ventricular (LV) non-compaction [12, 13]. Even though hypertrophic cardiomyopathy (HCM) is the most common cardiomyopathy type in the population [2], few studies have been conducted on the combination of myocarditis and HCM, highlighting the importance of further research in this area.

This systematic review aimed to clarify the incidence of myocarditis in HCM and to analyze its influence on the clinical presentation and outcome of patients with HCM.

## 2. Materials and Methods

The present systematic review was conducted in accordance with the Preferred Reporting Items for Systematic Reviews and Meta-Analyses (PRISMA) guidelines. The PICO (**p**atient, **i**ntervention, **c**omparison, **o**utcome) strategy was used to search for articles:

- Patient: patients over 18 years old with HCM and myocarditis

- Intervention: none (active monitoring)

- Comparison: patients with HCM without myocarditis

- Outcome: clinical deterioration in patients with HCM (death, heart transplantation, onset/decompensation of heart failure, onset/progression of rhythm disturbances).

### 2.1 Data Sources

The literature search focused on identifying articles in English and Russian published until November 2023 in the following medical literature databases: PubMed and the Scientific Electronic Library eLIBRARY.ru.

### 2.2 Inclusion Criteria

The inclusion criteria encompassed original studies reporting the rate, clinical features, and impact of myocarditis on prognosis in patients with HCM and clinical cases of patients with HCM and verified myocarditis.

**Fig. 1.**
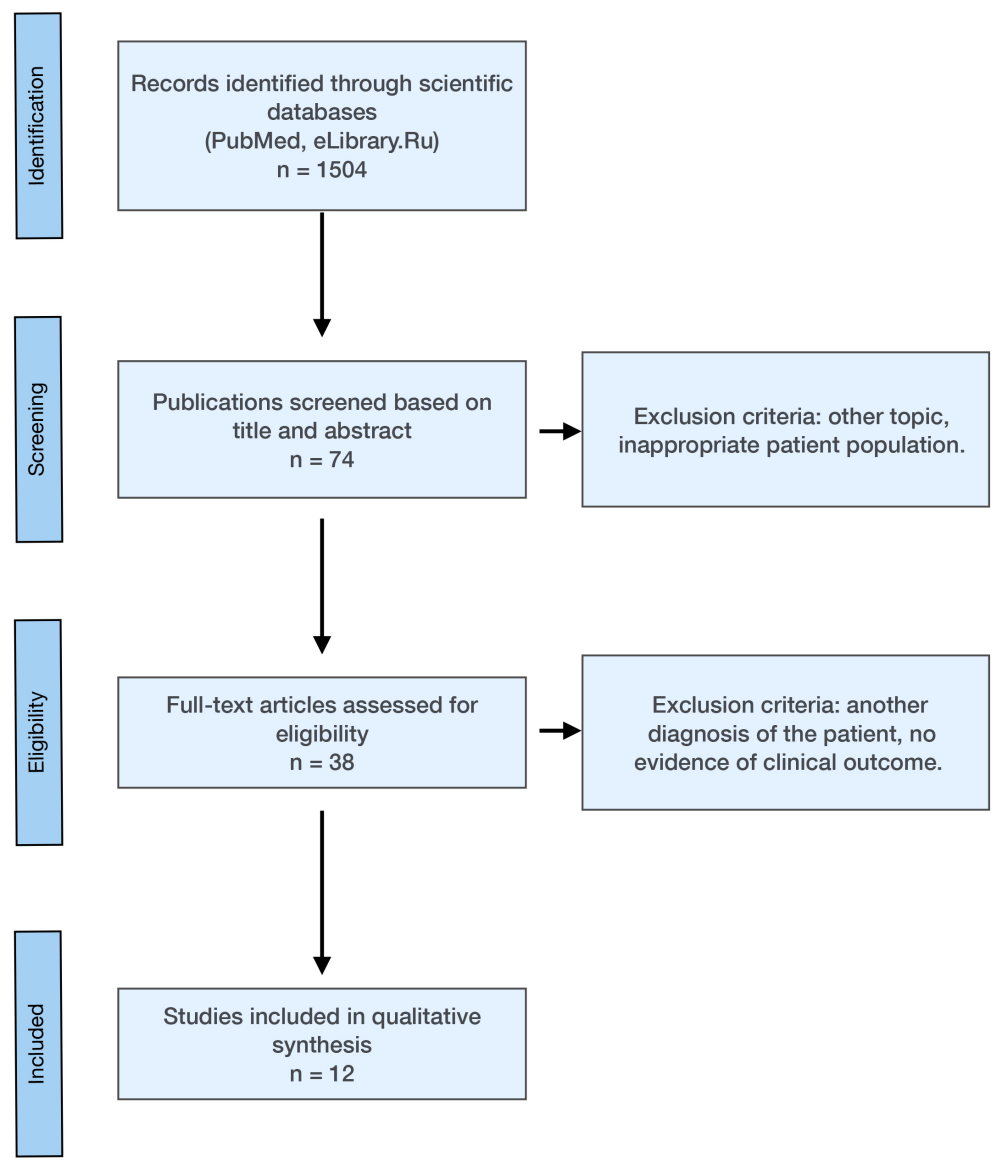
**PRISMA flowchart of database searches**. PRISMA, Preferred Reporting Items for Systematic Review and Meta-Analysis Protocols.

The search results yielded 1504 articles. We excluded articles that did not include patients with HCM in combination with myocarditis, and 457 publications were selected for further analysis. The subsequent exclusion criteria were the lack of information on the impact of myocarditis on the disease progression and clinical outcomes. In the end, twelve papers were included in the systematic review: three original studies and nine clinical cases (Fig. 1). The study protocol was registered in the International Prospective Register of Systematic Reviews PROSPERO (CRD42024499672).

### 2.3 Search Strategy

The following keywords were used to identify relevant papers through searches of the article title or abstract: [“hypertrophic cardiomyopathy” OR “HCM” OR “primary myocardial hypertrophy”] AND [“myocarditis” or “inflammat*”].

Articles were selected from the aforementioned databases in three stages. Initially, the titles of the articles were analyzed. Papers that did not meet the inclusion criteria for the review were excluded. Secondly, we analyzed the abstracts of the selected articles. Again, articles that did not meet the inclusion criteria were excluded. Thirdly, we analyzed the full text of the articles included in the review based on the results of the first two stages. Articles that did not meet the inclusion criteria were again excluded.

In total, 1131 articles were found in PubMed that met the search criteria. After reading the titles, 676 articles were excluded, and a further 36 were excluded after reading the abstracts. Thirty-four articles were selected for the full-text study, 12 of which were included in the research.

We found 373 articles that met the search criteria in the scientific electronic library eLIBRARY.ru. After reading the titles and removing duplicates, we excluded 369 articles from the analysis. We also studied the abstracts of four articles, but these were excluded from the analysis after reading the full paper. Thus, the study did not include articles from the scientific electronic library eLIBRARY.ru.

### 2.4 Assessment of Risk Bias 

Two review authors (NS and PC) worked independently to judge the risk of bias in each domain and the applicability of results using the “The Risk Of Bias In Non-randomized Studies of Exposures” (ROBINS-E) tool [14]. Study 1 [15] was judged as ‘low’ on all domains relating to bias or applicability except for concerns about uncontrolled confounding in domain 1 (D1), and the overall judgment for that study was a low risk of bias. Studies 2 and 3 [6, 16] were judged as ‘unclear’ because of some concerns in domains 1, 2, and 5, but no domains were at high or very high risk of bias.

**Fig. 2.**
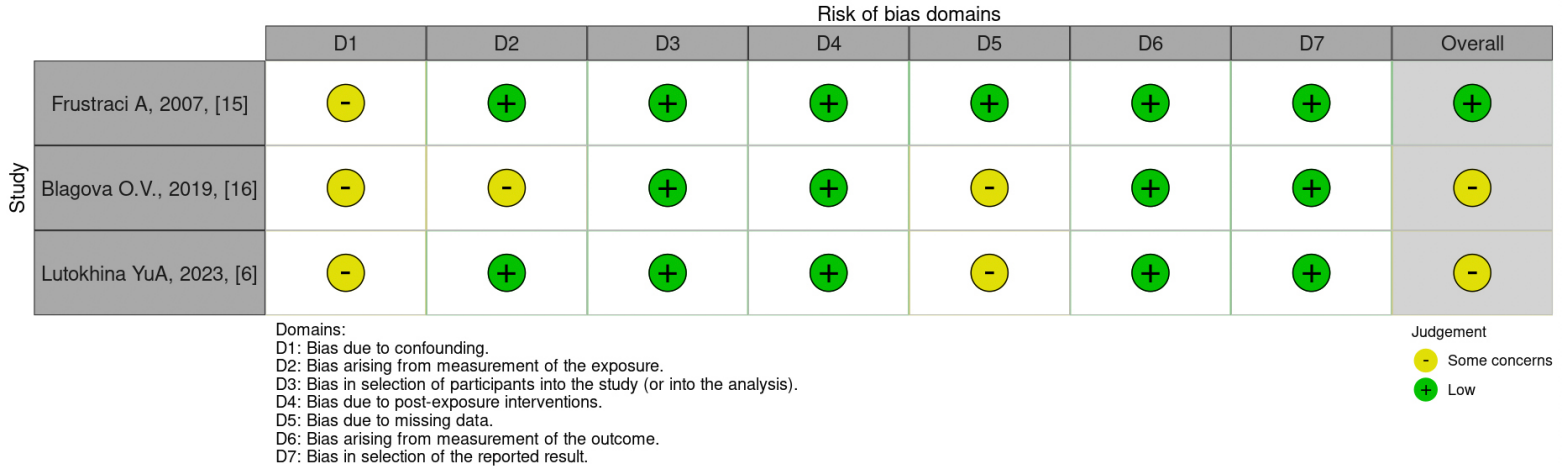
**Cochrane risk of bias results**.

Fig. 2 presents the results of the detailed evaluations. The included studies had a moderate overall quality.

## 3. Results

We selected three original articles and nine clinical cases for the final review. The results of the original study analysis are summarized in Table 1 (Ref. [6, 15, 16]).

**Table 1.** **The results of the original studies analysis**.

Study	Author, year, country	Patients	Characteristics	Diagnosis of myocarditis	Incidence of myocarditis	Outcomes	Conclusion
Myocarditis in hypertrophic cardiomyopathy patients presenting acute clinical deterioration [15]	Frustaci *et al*., 2007, Italy	119 patients with HCM	42 patients in a state of decompensation:	All patients underwent LV-EMB with histology, IHC, and PCR for cardiotropic viruses	23.5% of patients with HCM, and 50% virus-positive myocarditis	Group A1 with myocarditis: 46%: progressive recovery of heart contractility; 54%: progression to end-stage heart failure	Myocarditis, especially viral, is a common cause of acute clinical deterioration in HCM (heart failure progression and worsening of ventricular arrhythmias)
		Control group: 50 patients with mitral stenosis	Group A1: 20 patients with heart failure progression;		65.5% of patients with heart failure progression (group А1)	Group A2 with myocarditis: 53%: reduction of ventricular arrhythmias;	
			Group А2: 22 patients with aggressive ventricular arrhythmias		68.2% of patients with arrhythmias (group А2)	47%: persistent arrhythmias, onset of contractile dysfunction. All these patients received an ICD and were included on a heart transplant list	
			Group В: 77 clinically stable patients		0% of clinically stable patients (group В)	Virus-positive patients with myocarditis had a worse outcome compared with virus-negative patients	
					0% of the control group	All Group B patients remained clinically stable	
Syndrome of primary myocardial hypertrophy: clinical and morphological, genetic diagnostics and comparison of sarcomerial variants of cardiomyopathy and its phenocopy [16]	Blagova *et al*., 2019, Russia	55 patients with primary myocardial hypertrophy	28 patients with isolated HCM,	Myocardium pathological study, assessment of anti-cardiac antibodies, viral genome detection in the blood/myocardium by PCR, cardiac MRI	46.7% of all patients with HCM, and 73.3% virus-positive myocarditis	There are no mortality data	Myocarditis lowers LVEF in patients with HCM
			10 patients with a combination of HCM and noncompaction myocardium			Patients with HCM and myocarditis exhibited decreased myocardial contractility	
Prevalence of myocarditis and its contribution to the course of primary myocardial hypertrophy [6]	Lutokhina *et al*., 2023, Russia	100 patients with primary myocardial hypertrophy	68 patients with HCM	Myocardium pathological study (n = 29), IHC, assessment of anti-cardiac antibodies (n = 43), free light chain level (n = 10), myocardial scintigraphy (n = 5), cardiac MRI (n = 31)	31% in all patients with HCM, and 33.3% virus-positive myocarditis	Mortality in HCM with concomitant myocarditis: 33.3%	Myocarditis leads to heart failure progression, worsening ventricular arrhythmias, and increases the risk of sudden cardiac death
						Mortality in isolated HCM: 6.4%	Immunosuppressive therapy in myocarditis can stabilize the condition of patients
						Patients who received immunosuppressive therapy show a decrease in the functional class of heart failure and the number of ventricular extrasystoles	

HCM, hypertrophic cardiomyopathy; LV-EMB, left ventricular endomyocardial biopsy; IHC, immunohistochemistry; ICD, implantable cardioverter-defibrillator; LVEF, left ventricular ejection fraction; PCR, polymerase chain reaction; MRI, magnetic resonance imaging.

A substantial study by Italian researchers, overseen by Frustaci *et al*. [15], encompassed 119 patients diagnosed with HCM (69 male/50 female; mean age 41 ± 8). Of these, 42 were found to be in a state of decompensation, while 77 were deemed clinically stable [15]. A total of 20 of the 42 patients in the unstable group (group A1) exhibited progressive exacerbation of heart failure symptoms (an increase by at least one New York Heart Association (NYHA) class) with deterioration of systolic function, while 22 (group A2) demonstrated multiple episodes of ventricular arrhythmias (Lown grade 4b). All patients underwent LV endomyocardial biopsy (EMB) and gene analysis of major sarcomeric proteins. Fifty patients with mitral stenosis who underwent intraoperative myocardial biopsy during mitral valve prosthesis were selected as a control group. There were no signs of myocarditis in any patients in the control group.

The EMB revealed the presence of activated CD45RO+ T lymphocytes (≥14/mm^2^), disarray, and focal necrosis of adjacent severely hypertrophied myocytes in 28 of 42 decompensated patients (13 from group A1; 15 from group A2), consistent with active myocarditis. There were no signs of myocarditis in any clinically stable patient. The viral genome was detected in 14 of the 28 patients with myocarditis. It was noted that virus-positive patients had a worse outcome compared with virus-negative patients in the HCM–myocarditis group. This confirms the association of an adverse prognosis with viral persistence in the myocardium. Furthermore, a review of the medical histories of patients with HCM revealed that flu-like symptoms were frequently documented several weeks to months before the deterioration of their clinical condition. Moreover, patients underwent ventriculography. A left ventricular aneurysm was found in 36% of patients with HCM and myocarditis, which could contribute either to cardiac function deterioration or to the occurrence of ventricular arrhythmias. The authors believe that a combination of inflammatory infiltration of the myocardium with areas of intense myocytolysis with elevated intraventricular pressure promoted left ventricular aneurysm formation. Coronary arteries were normal in all patients.

As demonstrated by Frustaci *et al*. [15], myocarditis, particularly of a viral nature, was identified as a frequent cause of acute clinical deterioration in HCM. The evidence of high cardiotropic virus detection in the myocardium in HCM suggests an increased susceptibility to viral infection by patients with this cardiomyopathy.

Russian scientists reached similar conclusions in a study that included 100 patients with primary myocardial hypertrophy (52 males/48 females; mean age 51.5 ± 15.7), 68 of whom were patients with HCM, including 21 patients (31%) with concomitant myocarditis [6]. The cardiotropic viral genome was detected in the myocardium of seven patients with myocarditis. According to the results of the study, patients with HCM and concomitant myocarditis had a significantly higher functional class of heart failure and lower ejection fraction (EF) than patients with isolated HCM. Myocarditis was associated with worsening conditions after viral infection and increased titers of anti-cardiac antibodies in the blood. Patients with HCM and myocarditis also had a more than three times higher mortality rate (33.3% vs. 6.4%, *p* = 0.01). The study noted that seven patients experienced fatal outcomes. Of these, three deaths occurred due to heart failure progression, one death occurred due to ventricular arrhythmias, two deaths occurred due to acute cerebrovascular accident, and in one case, the underlying cause of death could not be ascertained. This finding underscores the pivotal role of myocarditis in the adverse outcomes of patients with HCM. Another notable trend is the stabilization of patients who received immunosuppressive therapy: a decrease in the functional class of heart failure and the number of ventricular extrasystoles. The results of this study confirm that myocarditis significantly contributes to the clinical picture in patients with HCM. Myocarditis has been shown to lead to heart failure progression, worsening ventricular arrhythmias, and increased risk of sudden cardiac death.

Another study by Russian researchers presented similar results [16]. The study comprised 55 patients diagnosed with primary myocardial hypertrophy (29 male/26 female; mean age 48.2 ± 17.0), 28 of whom were diagnosed with HCM. Fifteen patients with HCM underwent a myocardial morphological study. Myocarditis was verified in 46.7% of patients with HCM: in 4 patients with isolated HCM and three patients with a combination of HCM and LV non-compaction. The viral genome was detected in the myocardium of 11 patients with HCM (73.3%). The authors emphasize that the decrease in EF in patients with HCM results from myocarditis. The high frequency of concomitant myocarditis among patients with HCM and LV non-compaction suggests that genetically impaired myocardium has an increased vulnerability to myocarditis of viral etiology.

Consequently, the three original studies demonstrated a high incidence of myocarditis in patients with HCM and its negative impact on the primary disease and prognosis.

The following section will provide a concise overview of clinical cases featuring a combination of HCM and myocarditis:

1. A 32-year-old patient with obstructive HCM and heart failure (NYHA, class II) experienced frequent episodes of ventricular tachycardia, with a reduction in ejection fraction from 75% to 32%. After four weeks of metoprolol treatment, the symptoms of heart failure progressed to NYHA class III [17]. Blood tests revealed eosinophilia (up to 1050/mm^3^) and increased plasma eosinophil cationic protein levels (288 ng/mL). The EMB findings suggested hypersensitive myocarditis. Consequently, the beta-blocker was discontinued, and prednisolone therapy was initiated. As a result, the ventricular arrhythmias disappeared, systolic function recovered completely (EF - 78%), and eosinophil levels in the blood decreased to 60/mm^3^. Therefore, acute clinical deterioration, systolic function decrease, and ventricular arrhythmias in the patient with HCM were caused by hypersensitive myocarditis, probably induced by metoprolol.

2. A 30-year-old man with a family history of HCM was admitted to the hospital with continuous chest pain and profuse night sweats [18]. There was a fever of up to 38 °C for two days. An electrocardiogram (ECG) showed ST-segment elevation in leads I and aVL and ST-T changes in leads II, III, aVF, and V1-V6. Coronary angiography demonstrated no significant stenoses. Furthermore, elevated troponin I (106 ng/mL), creatine phosphokinase-MB (206.5 ng/mL), and myoglobin (502 ng/mL) were detected in the blood serum of the patient. Conversely, all blood and sputum cultures yielded negative results. TORCH (toxoplasmosis, other agents, rubella, cytomegalovirus, herpes simplex virus) screening revealed positive herpes simplex virus (HSV), rubella virus (RV), and cytomegalovirus (CMV) IgG, but all had negative IgM titers, suggesting previous infection. Echocardiography (EchoCG) showed biatrial dilatation, diffuse LV hypokinesia with an EF of 44%, asymmetric left ventricular hypertrophy (LVH) without LV outflow tract obstruction (interventricular septum (IVS): 17 mm), and moderate pericardial effusion. Cardiac MRI confirmed IVS hypertrophy (IVS: 22 mm). Subsequent DNA testing of the patient and his family members revealed a variant of uncertain significance.

The patient’s condition and laboratory parameters improved significantly after treatment with methylprednisolone, diuretics, and beta-blockers. On the 12th day of hospitalization, the patient was discharged. According to the EchoCG data, the LVH persisted one month after discharge; however, LV contractility recovered after myocarditis resolution.

In this clinical case, myocarditis led to the diffuse hypokinesis of the LV, which caused acute decompensation in the HCM patient.

3. A 45-year-old man was admitted in a coma, with a body temperature of 36.2 °C, pulse 35/min, and blood pressure (BP) 90 mmHg [19]. The day before admission, he suffered from fever and epigastric pain. Intrathoracic pain developed the next day. Blood tests showed leukocytosis up to 37,800/mm^3^, elevation of erythrocytes up to 72 mm/h and C-reactive protein (CRP), increase in transaminases, lactate dehydrogenase, blood urea nitrogen (BUN) and creatine phosphokinase (CPK) up to 386 IU/L. An ECG illustrated bradycardia, inverted T waves in II, III, and aVF, V2-V6, ST-segment depression in V2–V6, and polymorphic ventricular extrasystoles. These findings suggest the presence of myocardial ischemia or hypertrophy and multiorgan failure due to cardiogenic shock. The patient subsequently suffered a sudden cardiac arrest during the ECG recording, resulting in death. According to the autopsy data, the heart mass was 600 g. Concentric LVH was noted (IVS: 24 mm; posterior wall: 18 mm). Postmortem coronarography revealed no stenoses and occlusions of coronary arteries. Histological examination showed extensive and diffuse disarray of hypertrophied myocardial fibers, pronounced infiltration of mononuclear cells (mainly T-lymphocytes), interstitial edema, and necrosis of cardiomyocytes. The patient was diagnosed with HCM in combination with Fiedler’s myocarditis. Consequently, the patient exhibited symptoms of fulminant myocarditis, which resulted in acute heart failure, ventricular arrhythmia, and sudden cardiac death.

4. A 66-year-old woman with arterial hypertension and angina pectoris was hospitalized due to progressive dyspnea, weakness, and substernal chest pain that had irradiated to the left arm over the previous month [20]. Moreover, signs of HCM had been illustrated on an EchoCG three years previously. Upon arrival at the hospital, the BP was 92/70 mmHg, heart rate (HR) was 90 beats/min, body temperature 97.8 °F, and respiratory rate 20/min. On physical examination, there was a systolic murmur at the right upper sternal border without irradiation to the carotid arteries. An ECG showed an isolated inversion of T-waves in the aVL.

Coronaroventriculography revealed the absence of hemodynamically significant stenoses in the coronary arteries, a 45 mmHg gradient in the LV, a large aneurysm in the diaphragmatic wall, and akinesis of the anterolateral, apical, septal, and posterolateral walls of the LV. EchoCG showed asymmetric LVH and systolic anterior motion of the mitral valve (systolic anterior motion (SAM) syndrome) with LV outflow tract obstruction without severe systolic dysfunction. Tachycardia, hypotension, and oliguria persisted for several days. On the first day of hospitalization, the serum CPK level peaked at 387 U/I and continued for 7 days. The ECG revealed deep T-wave inversions in II, III, aVF, and V2–V6.

A rest and redistribution thallium study on the third day of hospitalization showed apical and infra-apical redistribution and a small fixed apical defect. EchoCG, on the seventh day of hospitalization, demonstrated improved LV systolic function, with an EF of 39%. A right ventricular EMB on day 13 revealed “borderline” myocarditis according to the Dallas criteria. An antimyosin scan showed 4+ activity involving the anterior, inferior, and septal walls of the LV, as well as possible uptake in the RV. On the 18th day of hospitalization, the patient was transferred to the rehabilitation center, where she was administered furosemide, captopril, and warfarin. There was a progressive improvement in symptoms over the following weeks, and a subsequent echocardiogram performed two months later revealed only a minor zone of dyskinesis in the LV apex; the EF was 71%, IVS thickness was 16 mm, and there were no signs of significant outflow obstruction (maximal gradient 16 mmHg) or SAM syndrome. The patient’s condition was considered to be acute myocarditis. The authors of the article concluded that myocarditis in a patient with HCM resulted in transient LV aneurism formation, serious hemodynamic disturbances up to shock, and complete recovery.

5. A 67-year-old female was urgently admitted to the hospital due to a sudden syncopal attack [21]. The patient was hemodynamically unstable on admission: BP 63/39 mmHg, HR 43 beats per minute, body temperature 36.7 °C. A physical examination revealed a systolic murmur in the area of the heart apex and crackles in almost the entire lung field. Chest X-ray showed cardiomegaly and pulmonary congestion. ECG revealed complete atrioventricular (AV) block and ST segment depression in I, II, III, aVL, aVF, and V3-V6 leads were registered. EchoCG revealed asymmetrical LVH (IVS 22 mm) with LV outflow obstruction (gradient 106 mmHg), SAM syndrome with severe mitral regurgitation, EF 65%. Serum levels of CPK, N-terminal prohormone of brain natriuretic peptide (NT-proBNP), and troponin T were elevated. Temporary pacing was installed due to a complete AV block, and cardiac catheterization was performed to clarify the cause of cardiogenic shock. It revealed a high-pressure gradient in the LV outflow tract - 120 mmHg, and no significant stenosis in the coronary arteries. An EMB of LV was performed, and signs of hypertrophied cardiomyocytes with interstitial fibrosis and edema with infiltration by mononuclear cells were described. The treatment resulted in a pressure decrease in the LV outflow tract to 20 mmHg, disappearance of AV block, and mitral regurgitation. The IVS thickness also decreased from 22 mm to 16 mm. However, a cardiac MRI performed on the 38th day after admission showed delayed IVS uptake. Seven days after discharge, the patient was readmitted to the hospital with complaints of dizziness. The ECG revealed an intermittent complete AV block again, and a dual-chamber pacemaker was implanted.

Therefore, acute myocarditis induced AV block in a patient with HCM.

6. A 50-year-old patient with HCM, infective endocarditis, and severe mitral regurgitation presented epigastric pain, dyspnea, orthopnea, and immeasurable BP [22]. Blood tests showed anemia, neutrophilic leukocytosis, elevated CPK level, and blood culture revealed a Gram-positive bacteria with a defective wall (L-form). EchoCG demonstrated signs of HCM, dilatation of both ventricles, dyskinetic IVS, akinesis of the apex and anterolateral wall, severe hypokinesia, and systolic dysfunction of the LV. An ECG revealed ventricular arrhythmias and disturbances in conduction.

The patient developed a paroxysm of AF, which led to the progression of heart failure. This condition was refractory to therapy and complicated by the development of ventricular tachycardia and ventricular fibrillation. The patient died.

A postmortem examination revealed severe asymmetric LVH. A morphological examination of the myocardium revealed inflammatory infiltrates rich in eosinophils, foci of myocytolysis, HCM-specific changes in cardiomyocytes, and signs of endocarditis.

The patient’s condition was considered hypersensitive myocarditis, which led to acute heart failure and sudden arrhythmic death.

7. A 65-year-old female patient was admitted to the hospital for treatment of esophageal varix due to cirrhosis associated with hepatitis C and complained of sudden chest pain and dyspnea [23]. The patient had no previous history of cardiac disease. On admission, the patient exhibited a blood pressure of 116/76 mmHg and a pulse rate of 89 beats per minute; during auscultation, a systolic murmur and moist wheezes in both lungs were noted. Blood tests demonstrated increased troponin T (2.21 mg/L) and CRP (81.1 µg/L). An ECG showed a right bundle block, ST-segment elevation, and pathologic Q in the anterolateral leads. EchoCG revealed akinesis of the anteroseptal and anterior LV segments, all segments below the papillary muscle, asymmetric LVH with LV outflow obstruction (max Hg 120 mmHg), and severe mitral regurgitation (SAM syndrome). The ventriculography results coincided with the EchoCG findings; the coronary arteries were intact.

During the administration of disopyramide and furosemide, there was a reduction in the severity of the patient’s chest pain and dyspnea. On the next day, a single photon emission computed tomography (SPECT) was performed, and a perfusion defect was detected in the areas of LV akinesis. EchoCG, performed on day 4, showed a reduction in the severity of LV outflow tract obstruction, absence of SAM syndrome, and improved LV contractility. Then, the resolution of the ST-segment elevation and recovery of R wave voltage in anterolateral leads were registered on the ECG. The EMB was performed on day 8: the picture corresponded to borderline myocarditis according to the Dallas criteria. The patient was discharged. An EchoCG on day 15 and 4 months later showed no LV contractility abnormalities and no obstruction in the outflow tract, but asymmetric LVH typical of HCM persisted. Repeated SPECT one month later revealed no areas of perfusion defect, and the EF increased up to 60%.

This clinical case demonstrates the development of severe heart failure with pulmonary edema and transient LV obstruction in a patient with HCM due to acute myocarditis. The authors hypothesize that the myocarditis may have been caused by persistent hepatitis C virus (HCV) infection.

8. A patient, aged 39, presented with symptoms including fatigue, fever, dyspnea, and palpitations from exertion [24]. A review of the patient’s family history revealed a significant event: his mother had died suddenly at the age of 55. The patient was admitted to the hospital. Beta-blockers were administered with a positive effect. Three years after discharge, the patient died suddenly. An autopsy revealed obstructive HCM (IVS: 32 mm; posterior wall: 22 mm) with extensive myocardial fibrosis; the heart mass was 700 g. Numerous patchy fibrosis areas of various sizes were observed in the LV walls. Microscopic examination showed severe fascicular disorganization of hypertrophied myocardial fibers, granulation tissue with many capillaries, and lymphocytic infiltrates. Pericardial fibrosis was also described. Thus, the patient had congenital HCM as well as myopericarditis. The authors suggest that the latter triggered the progression of cardiomyopathy.

9. A 47-year-old patient with apical HCM was hospitalized with severe biventricular failure requiring inotropic support [25]. A large number of eosinophilic infiltrates were noted in the explanted heart. The authors hypothesize that eosinophilic myocarditis could be associated with dobutamine infusion therapy. However, the likelihood of myocarditis being the direct cause of decompensation is considered to be higher, given that the apical form of HCM itself is usually stable and does not typically lead to refractory heart failure.

Table 2 (Ref. [17, 18, 19, 20, 21, 22, 23, 24, 25]) summarizes the clinical case analysis results.

**Table 2.** **The results of the clinical cases analysis**.

Study	Author, year, country	Patient (age, years)	Diagnosis of myocarditis	Characteristics	Immunosuppressive therapy	Outcomes	Conclusion
Hypersensitivity myocarditis induced by beta-blockers: an unexpected cause of abrupt deterioration in hypertrophic cardiomyopathy [17]	Frustaci *et al*., 2007, Italy	F (32)	LV-EMB.	Obstructive HCM, heart failure (NYHA, class II), hypersensitive myocarditis, which was induced by beta-blockers.	Prednisolone	Complete recovery of cardiac function.	Hypersensitive myocarditis promoted an abrupt deterioration in cardiac function.
Myocarditis combined with hypertrophic cardiomyopathy: a case report [18]	Wang *et al*., 2021, China	М (30)	TORCH screen, cardiac MRI.	HCM and myocarditis.	Methylprednisolone	LVEF recovery.	Myocarditis led to an LVEF decrease and the progression of heart failure.
Sudden cardiac death from hypertrophic cardiomyopathy and acute idiopathic (Fiedler’s) myocarditis: autopsy report [19]	Takata *et al*., 1993, Japan	М (45)	Autopsy.	HCM and Fiedler’s myocarditis.	—	Sudden cardiac arrest and death.	Myocarditis caused acute heart failure, ventricular arrhythmias, and sudden cardiac death in the patient with HCM.
Transient left ventricular aneurysm in a patient with hypertrophic cardiomyopathy and myocarditis [20]	Fisher *et al*., 1993, USA	F (66)	Right ventricular biopsy, antimyosin scan.	Obstructive HCM, myocarditis.	—	Full recovery of LVEF, persisted LVH, reduction in the severity of LV outflow tract obstruction.	Myocarditis caused LV dysfunction, transient LV aneurysm formation, hemodynamic disturbances up to shock, and subsequent complete recovery.
Cardiogenic shock due to left ventricular outflow obstruction and complete atrioventricular block in a patient with hypertrophic cardiomyopathy with acute myocarditis [21]	Kusumoto *et al*., 2012, Japan	F (67)	LV-EMB, cardiac MRI, immunology laboratory tests.	Obstructive HCM, myocarditis, complete AV blockade.	—	Decrease in LV pressure gradient, IVS thickness reduction, the disappearance of mitral regurgitation, and AV blockade.	Myocarditis promoted disturbances in conduction up to complete AV blockade.
						AV blockade reoccurred one week after discharge. A dual-chamber pacemaker was implanted.	
Hypersensitivity myocarditis, a surprising diagnosis. Case report [22]	Butcovan *et al*., 2006, Romania	М (50)	Autopsy.	HCM, infective endocarditis, hypersensitive myocarditis, conduction disorders.	—	The patient suddenly died due to heart failure decompensation.	Hypersensitivity myocarditis resulted in a fatal outcome.
Dynamic outflow obstruction due to the transient extensive left ventricular wall motion abnormalities caused by acute myocarditis in a patient with hypertrophic cardiomyopathy: reduction in ventricular afterload by disopyramide [23]	Sakai *et al*., 1999, Japan	F (65)	SPECT scan, LV-EMB.	Obstructive HCM and myocarditis.	—	Chest pain and dyspnea decreased.	Myocarditis caused acute heart failure with pulmonary edema in a patient with HCM.
				Liver cirrhosis associated with hepatitis C.		Full recovery of LVEF, absence of LV outflow tract obstruction but asymmetric LVH persisted.	HCV may be an etiologic agent of myocarditis in HCM.
						SPECT one month later revealed no areas of perfusion defect.	
Hypertrophic obstructive cardiomyopathy with extensive myocardial fibrosis: case report with autopsy [24]	Hirama *et al*., 1985, Japan	М (43)	Autopsy.	Obstructive HCM and myocarditis.	—	The patient died.	Myocarditis led to heart failure decompensation and sudden cardiac death.
Hypersensitivity myocarditis complicating hypertrophic cardiomyopathy heart [25]	Butany *et al*., 2004, Canada	F (47)	Histological examination of the myocardium.	HCM and eosinophilic myocarditis.	—	Significant decrease in LVEF.	Eosinophilic myocarditis caused an abrupt deterioration in cardiac function.

HCM, hypertrophic cardiomyopathy; LV, left ventricle; LVH, left ventricular hypertrophy; IVS, interventricular septum; LV-EMB, left ventricular endomyocardial biopsy; AV, atrioventricular; LVEF, left ventricular ejection fraction; MRI, magnetic resonance imaging; SPECT, single photon emission computed tomography; HCV, hepatitis C virus; M, male; F, female; NYHA, New York Heart Association; TORCH, toxoplasmosis, other agents, rubella, cytomegalovirus, herpes simplex virus.

## 4. Discussion

A qualitative analysis of the results from original studies and a series of clinical cases revealed that the onset of myocarditis alongside a background of HCM promotes the worsening of clinical features and development of congestive heart failure decompensation, as well as the appearance or aggravation of heart rhythm disturbances. The manifestations of this condition, as observed in the reviewed studies, encompass a range of outcomes, including reduced EF and ventricular arrhythmias, as well as the formation of LV aneurysms and the development of AV blocks, which can ultimately result in acute heart failure. The original studies provide compelling evidence that patients with isolated HCM demonstrate a significantly more favorable course and prognosis than those with myocarditis. Furthermore, the persistence of the viruses within the myocardium exerts a negative impact on the prognosis.

It has been established that viruses can induce a chronic inflammatory response in the myocardium. Chronic myocarditis provides a basis for triggering the hypertrophic growth of cardiomyocytes [26]. The established role of the HCV in the pathogenesis of chronic myocarditis should be mentioned separately. Recent study has emphasized the significance of HCV infection in patients with myocarditis, as well as in those with dilated cardiomyopathy and HCM [27]. There is evidence of HCV infection in patients with HCM: HCV RNA has been detected in the myocardium of patients, and anti-HCV antibodies are present in the serum. This observation suggests the potential for HCV to replicate in the heart, contributing to the development of hypertrophy [28]. Furthermore, a statistical correlation has been identified between elevated levels of HCV antibodies and both HCM and dilated cardiomyopathy [29]. Moreover, positive antibodies are even more prevalent in patients with HCM than those with dilated cardiomyopathy. Based on the study by Omura T *et al*. [30], it can be assumed that the expression of the HCV core gene (the main component of the viral nucleocapsid) could lead to progressive morphological and functional changes, eventually leading to the development of inflammation and histological signs consistent with HCM.

In addition to the pivotal role of viral infection in the pathogenesis of HCM, evidence exists from studies demonstrating a correlation between cardiac sarcoidosis and HCM. This association is shown by the Matsumori A. study, which suggests that cardiac sarcoidosis may cause myocardial changes that mimic HCM [31]. This finding reinforces that HCM and the myocardial inflammatory response, including autoimmunity, are closely related.

Individual clinical cases and one of the original studies have demonstrated the significance of diagnosing myocarditis in HCM and the role of immunosuppressive therapy (IST) in improving outcomes [6, 17, 18]. Fatal outcomes were only reported in cases where IST was not mentioned, and these patients were most likely not receiving IST. This finding has important clinical implications because, in real practice, the diagnosis of myocarditis is not always followed by appropriate pathogenetic therapy.

It is important to note that the analysis of the papers presented in this review has confirmed that a genetically compromised myocardium is more susceptible to inflammation. The following discussion will explore the potential for molecular genetic mechanisms to explain the elevated predisposition of patients suffering from HCM to myocarditis. In the majority of cases, HCM is caused by mutations in genes that encode sarcomeric proteins, including *MYH7* (β-myosin heavy chain), *MYBPC3* (myosin-binding protein C), and *TNNT2* (cardiac troponin T). Consequently, calcium homeostasis is disrupted, and the sensitivity of the myofilaments to calcium ions is enhanced. The force of systolic sarcomere contractions increases while diastolic relaxation decreases. As a result, there is an increased demand for ATP by the cardiomyocytes, the impaired transmission of regulatory signals within the cells, and subsequent activation of the hypertrophic growth program [32].

Some mutations associated with HCM may increase the susceptibility of the myocardium to inflammatory processes. In the study by Vakrou *et al*. [33] on mouse models, it was demonstrated that a mutation in the *MYH7* gene increases the activity of inflammasomes, which contribute to the activation of the inflammatory cascade by activating inflammatory caspases and interleukin (IL)-1β. At the same time, the clinical presentation in these mice was marked by severe symptoms, including arrhythmias and heart failure [33]. Notably, the Lutokhina study, which was incorporated into our review, revealed a higher prevalence of mutations in the *MYH7* gene among patients diagnosed with myocarditis in combination with HCM compared to those with isolated HCM [6].

The analysis of a cohort of patients with HCM and a control group of 17 healthy subjects by Kuusisto *et al*. [34] revealed that there is a weakly expressed myocardial and general inflammatory response in HCM due to the sarcomeric mutation (*TPM1-D175N*). The presence of myocardial inflammatory cell infiltration, elevated myocardial nuclear NF-κB activity, and circulating inflammatory cytokines indicate inflammation in patients with HCM. The activation of NF-κB results in a proinflammatory phenotype characterized by the upregulation of TNF-α, which activates inflammatory cell and fibroblast infiltration, leading to the formation of perivascular fibrosis in the myocardium [34].

The study by Li *et al*. [35] indicates that the expression of the protein genes *YTHDC1* and *IGFBP-3* is increased in individuals diagnosed with HCM. Increased *IGFBP-3* activity results in myocardial fibrosis by activating epithelial–mesenchymal transition (a reversible process in which epithelial cells transform into fibroblast-like cells). Moreover, myocardial infiltration by immune cells and elevated levels of TNF-α and IL-6 gene expression were observed. The increased expression of *YTHDC1* results in reduced mitophagy activity and slower energy metabolism, which may contribute to the potentiation of oxidative stress and inflammation [35].

In a study by Lynch *et al*. [36] on mice with dilated cardiomyopathy with the c*MyBPC*(t/t) genetic variant, significantly higher levels of activated lymphocytes and proinflammatory M1 macrophages were observed in mice with the *MyBPC3* mutation than in those without. Moreover, a statistically significant difference was observed in the mutation rates between the two groups (14.8 ± 1.4% vs. 6.5 ± 1.4%, *p* = 0.002) and (10.3 ± 1.2% vs. 3.4 ± 0.8%, *p* = 0.0009). Thus, this molecular genetic mechanism is highly likely implicated in the pathogenesis of HCM, given the established association between *MyBPC3* mutations and this specific manifestation of cardiomyopathy. However, it should be noted that such mutations can also occur in association with other cardiomyopathy phenotypes [36].

Thus, several different molecular genetic mechanisms may contribute to the development of inflammation in cardiomyopathies, particularly in HCM. The presence of mutations in specific genes has been demonstrated to elevate the risk of inflammation in HCM. At the same time, myocarditis in the context of cardiomyopathy increases the risk of heart failure, decompensation, and arrhythmias, including sudden cardiac death. Consequently, the concurrence of myocarditis and HCM cannot be considered a mere coincidence, as genetically compromised myocardium is a favorable background for the onset of inflammation.

### Limitations

The present review has convincingly demonstrated the high incidence of myocarditis in HCM and its significant contribution to the clinical course of this cardiomyopathy. Nevertheless, it is important to acknowledge the limitations of the present systematic review. Despite the great importance of the inflammatory process in HCM, only three original studies address this issue. These studies have a moderate to high confidence level, but their design is significantly different, making it impossible to perform a meta-analysis. We have augmented the review with several clinical cases to provide a more comprehensive overview of the interplay between myocarditis and HCM. However, these are also quite heterogeneous, so they cannot be completely standardized. Nevertheless, these cases reflect the difficulty of diagnosing myocarditis in HCM and the importance of its management in specific clinical examples. Moving forward, conducting randomized trials to evaluate the efficacy of IST for myocarditis in HCM appears to be a pivotal endeavor.

## 5. Conclusions

The prevalence of myocarditis in patients with HCM is significant, with estimates ranging from 23.5% to 46.7%. The findings of this study indicate that the presence of concomitant myocarditis in patients with HCM is associated with an increased risk of heart failure progression, worsening of ventricular arrhythmias, and sudden cardiac death. Therefore, the pathogenetic treatment of myocarditis in HCM is recommended, as evidence exists that immunosuppressive therapy can reduce the severity of heart failure and arrhythmias and improve prognoses.

## Availability of Data and Materials

All data reported in this paper will also be shared by the lead contact upon request.

## Supplementary Material

Supplementary material associated with this article can be found, in the online version, at https://doi.org/10.31083/RCM28234.
